# Adaptation to Telehealth of Personalized Group Visits for Late Stage Diabetic Kidney Disease

**DOI:** 10.34067/KID.0000000000000301

**Published:** 2023-11-14

**Authors:** Connor Drake, Abigail Rader, Christie Clipper, Malia Haney, Dominique Bulgin, Blake Cameron, Tara Kinard, Devdutta Sangvai, Susan Tomso, Ralph Snyderman

**Affiliations:** 1 Department of Population Health Sciences, Duke University School of Medicine, Durham, North Carolina; 2 Center for Personalized Health Care, Duke University School of Medicine, Durham, North Carolina; 3 Center of Innovation to Accelerate Discovery and Practice Transformation, Durham Veterans Affairs Health Care System, Durham, North Carolina; 4 College of Nursing, The University of Tennessee, Knoxville, Tennessee; 5 Department of Medicine, Duke University School of Medicine, Durham, North Carolina; 6 Population Health Management Office, Duke University Health System, Durham, North Carolina; 7 Digital Strategy Office, Duke University Health System, Durham, North Carolina

**Keywords:** group visits, disease care management, personalized health planning, CKD, primary care, care management redesign, patient-centered care, telehealth

## Abstract

**Key Points:**

- Improving late stage diabetic kidney disease care requires adapting evidence-based, self-management programs for telehealth delivery.
- We adapted and pilot-tested a telehealth approach and found it to be feasible. Preliminary data suggested it improved relevant health and patient-recorded outcomes.

**Background:**

The coronavirus disease 2019 pandemic resulted in an unprecedented shift in the delivery of outpatient medical care, including the rapid transition of services from in-person to telehealth. We adapted an evidence-based personalized health planning group visit care model traditionally offered in-person to telehealth to support the care of patients with type 2 diabetes mellitus (T2D) and CKD. Despite the need to leverage telehealth technologies to better support self-management for patients with CKD, scant evidence exists on how to do so.

**Methods:**

We conducted prospective adaptations of in-person evidence-based group visit model for telehealth delivery for patients with CKD and T2D. Intervention adaptations are reported using the Framework for Reporting Adaptations and Modifications–Expanded taxonomy. The adapted virtual group visit care model was pilot-tested among adults with T2D and stage 3b or 4 CKD. Feasibility outcomes included recruitment, attendance, satisfaction, and self-reported goal progress. Clinical outcomes were evaluated using Wilcoxon signed-rank tests and included hemoglobin A1c, diastolic and systolic BP, body mass index, and eGFR.

**Results:**

Adaptation areas included outreach, visit format, educational materials design and access, staffing, and patient engagement strategies. 39% (43) of patients (110) contacted verbalized interest, and 58% (25) of those participated. 72% completed >6 group sessions. 68% of patients reported completing one or more health goals, with nutrition and physical activity being the most common. We observed a statistically significant improvement in hemoglobin A1c (*P* = 0.0176) 6 months postprogram participation.

**Conclusions:**

Adapting evidence-based interventions for telehealth delivery is challenging because of the risk of altering an intervention's core components responsible for observed benefits. We adapted an in-person group visit model for the care of T2D and CKD for telehealth delivery. The telehealth approach was feasible, and preliminary data suggested it improved relevant health and patient-recorded outcomes up to 6 months postprogram completion. The approaches used here may be applicable to the adaptation of other clinical programs for telehealth delivery.

**Podcast:**

This article contains a podcast at https://dts.podtrac.com/redirect.mp3/www.asn-online.org/media/podcast/K360/2023_12_29_KID0000000000000301.mp3

## Graphical abstract

**Figure:**
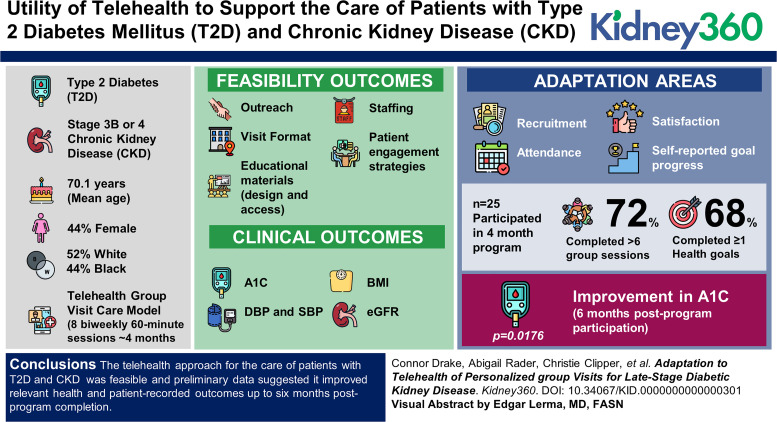

## Introduction

The coronavirus disease 2019 pandemic has accelerated the uptake of novel models of care delivered by telehealth, and this trend is likely to continue.^1^ This is particularly true for those with complex chronic illnesses with health self-management responsibilities including type 2 diabetes mellitus (T2D), cardiovascular disease, and CKD. Telehealth and virtual health care delivery modes can be efficient and contribute to increased patient satisfaction.^2^ However, best practices on how to adapt and implement evidence-based, in-person interventions for virtual delivery is lacking.

**Figure 1:**
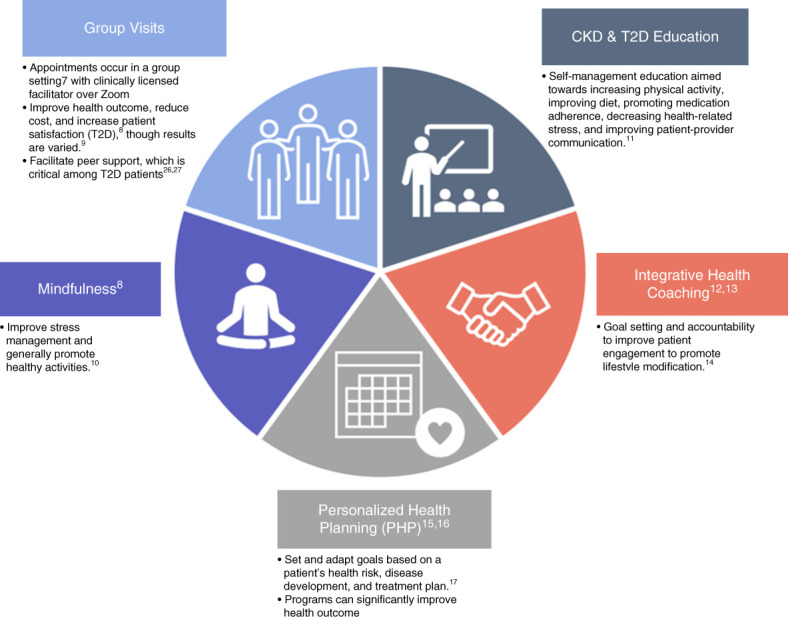
**Personalized group visit intervention description.** PHP, personalized health planning; T2D, type 2 diabetes mellitus.

Over 30 million people in the United States have CKD^3^ and that number is rising.^4^ CKD is of particular concern because of the high comorbidity rate with other conditions, such as cardiovascular disease and diabetes,^5,6^ and the corresponding high risk of mortality.^7,8^ Furthermore, approximately one in three adults with T2D have CKD,^9^ creating an additional self-management burden. While there are available therapies to mitigate these conditions, treatment adherence in CKD is suboptimal^10^ and hindered by often intersecting medical, behavioral, and social needs.^11^ Therefore, there is a critical need for comprehensive, evidence-based interventions to promote self-management skills among patients with CKD. Personalized health planning (PHP) within group visits, that is, personalized group visits, is one such approach to delivering comprehensive chronic disease care. Personalized group visits incorporate personal health plans and goals^12^ with a group-based format,^13,14^ integrative health coaching,^15,16^ mindfulness meditation,^17^ and self-care education. This approach (Figure 1) has the potential to improve health outcomes, enhance patient satisfaction, and reduce costs for chronic and complex conditions that involve interrelated medical, behavioral, and social needs.^13,18–22^ However, a systematic review of the effect of group visit interventions across different chronic conditions revealed a lack of testing in interventions specifically tailored to meet the unique needs of patients with CKD.^23^ Given evidence that the application of the PHP group visit model has benefits in management and prevention of T2D^24^ and prediabetes,^25^ we hypothesized that the model could be adapted to telehealth and improve outcomes for patients with CKD. Adaptations, or modifications to retain and improve effectiveness of interventions in real-world settings, are needed to support implementation.^26^ Because real real-world settings are different from trial conditions, adaptations of interventions to improve fit within existing practice patterns are necessary. Importantly, intervention adaptations rarely exist within a vacuum and are often made in response to a broader context within a clinical setting with the goal of preserving fidelity of components that benefit a patient.^27^ Adaptation of more complex self-management behavioral interventions are a topic of significant interest, particularly for managing intersecting chronic conditions such as T2D, CKD, and hypertension.^28^ Adapting interventions for populations at heightened cardiometabolic risk is critical to offer evidence-based telehealth programs to improve continuity of care and self-management support. The PHP group visit model was adapted to ensure educational modules, and coaching were tailored to the needs of those managing CKD, and T2D and could be delivered in a virtual (*i.e.*, synchronous audio and video telehealth) format.

The study herein describes the prospective adaptation of the PHP framework, originally developed for T2D, for use with CKD as part of a comprehensive approach and delivered through virtual conferencing. The pilot of this comprehensive program was designed to collect data on feasibility and preliminary clinical outcomes for adults with CKD and T2D. In doing so, this work provides insights into best practices for designing effective telehealth interventions to support CKD self-management as a complementary program to existing clinical care.

## Methods

### Group Visit Model

The telehealth group visit program included eight biweekly 60-minute sessions over approximately 4 months conducted over a Health Insurance Portability and Accountability Act-compliant video visit platform (Zoom) that had been integrated into existing electronic health record and patient portal systems (Epic Systems) with health education modules (Xealth). The group visit sessions were designed to align with guideline-concordant self-management education content related to CKD and chronic conditions: medication, nutrition, stress, exercise and sleep, and personal growth.^24^ The sessions were titled, “Your Health and CKD,” “Goal Setting and Your Personal Health Plan,” “What You Eat and Drink,” “Stress and Your Thoughts,” “Exercise and Sleep,” “Medicines and Your CKD,” “Developing Yourself and Your Relationships,” and “Moving Forward.” Participants completed a health risk assessment and consult with their health care provider to establish a therapeutic plan and set health goals. Streamlining this process, health goals were prepopulated based on guideline-concordant recommendations for self-management of CKD. Additional detail describing the goal-setting process through PHP is described elsewhere.^12^ Each session included mindfulness practice, and participants were provided access to digital and printed notebooks with educational content and self-management tools that corresponded to the topic covered. Patients were divided into two cohorts which independently met and completed the program facilitated by a health coach (facilitator) and cofacilitator. The facilitator held either a national board health coach certification or an integrative medicine health coach certification, along with clinical licensure as a registered nurse. Group visits were complemented by two one-on-one coaching sessions conducted telephonically throughout the program.

### Intervention Adaptation Tracking

Program adaptations were made to accommodate (*1*) the intersecting, social, behavioral, and medical needs of patients and their caregivers with CKD and T2D; (*2*) a telemedicine, virtual delivery; and (*3*) existing technologies and infrastructure specific to the program's context. We used Stirman's Framework for Reporting Adaptations and Modifications–Expanded^27^ to describe program modifications that were both proactive and planned and in reaction to unanticipated challenges. By doing so, the rationale and effect of adaptations can be assessed across different categories. These include when and how the modification(s) were made, whether it was planned, the decision-making process, and the nature and characteristics of the modifications with the goal of preserving fidelity to the needed evidence-based components.

**Figure 2:**
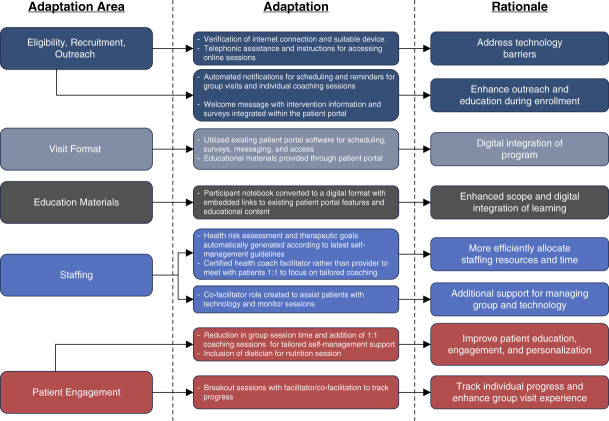
Overview of personalized group visit adaptation for telehealth delivery to patients with late stage diabetic kidney disease.

Consistent with best practices for tracking chronic illness intervention adaptations, we engaged with relevant stakeholders, investigators, and research staff and reviewed projects records and meeting minutes on the process of adapting the group visit program to telehealth delivery. Descriptive monitoring of modifications occurred across the following core intervention components of the PHP group visit model: (*1*) eligibility, recruitment, and outreach; (*2*) group and individual visit format; (*3*) self-care educational materials design and access; (*4*) staffing; and (*5*) patient engagement. Tracking of adaptations was used iteratively to identify strategies to overcome anticipated implementation barriers. See Figure 2 for additional detail.

### Pilot Study Setting and Data Collection

We performed a pilot cohort study using the adapted, virtual group visit program intervention on adult patients with both T2D and stage 3b or 4 CKD who were attributed to the accountable care organization managed by the Duke Population Health Management Office. We excluded patients with other non-CKD serious illness, blindness, or severe mental illness. The intervention was provided at no cost to participants, and caregivers (or other significant individuals) were permitted to participate. Participation was voluntary, and monetary incentives were not provided. Patients were invited through their EHR-enabled patient portals and received a follow-up call from study team members to screen and assess anticipated technology barriers.

Outcomes included attendance, satisfaction, acceptability, self-reported health behaviors, health goal progress, and clinical outcomes relevant to patients with heightened cardiometabolic risk. Satisfaction measures included whether the patient would participate again or recommend the program to others. Acceptability measures included questions related to the convenience of the program, comfort participating and sharing health information, perceptions of the health coach/facilitators, and a question on requested changes to the program/curriculum. Health goals were established and tracked between the third and final sessions and categorized on the basis of self-care areas of focus. The research team used a goal progress measure developed from previous studies^24^ to record progress for each patient and goal. We recorded the highest progress over the eight sessions, with achieved goal being the highest possible progress, followed by made progress, try again, did not address, and dropped goal. We assessed systolic BP, diastolic BP, hemoglobin A1c (HbA1c), eGFR, and body mass index. Clinical outcomes were obtained using retrospective chart review by a member of the study team (M Haney) with preintervention values acquired up to 6 months before intervention and up to 6 months postintervention. We collected data on patient satisfaction and general self-rated health using a survey on the basis of our previous research^24^ as well as validated instruments.^29–32^ To evaluate the change in outcome before and after the intervention, we used a Wilcoxon signed-rank test to report a two-sided *P* value and made no adjustment for multiple comparisons at baseline to 6 months after the intervention. The Wilcoxon signed-rank test was used for our matched-pair analysis because of the small sample size that precluded assuming a Gaussian distribution. The Duke University Health System Institutional Review Board reviewed this project and deemed it nonresearch. All analysis was conducted using Microsoft Excel and SAS On Demand for Academics.

## Results

Adaptations using the Framework for Reporting Adaptations and Modifications–Expanded taxonomy reported modifications from the previously validated in-person intervention. These adaptations spanned several focus areas including eligibility, recruitment and outreach, group and individual visit format, self-care educational materials design and access, staffing, and patient engagement. Noteworthy adaptations included the overall shift from a diabetes-focused in-person to a virtual format focused on patients with both T2D and CKD. In addition, the intervention eliminated the medical appointment component, having the program function as a complementary to rather than a replacement for traditional medical care. Of note, there was emphasis on ensuring patient confidence and literacy with technology during outreach, enrollment, and appointment reminders. A cofacilitator was used to assist patients with technical difficulties and group management. This focus on ensuring digital health literacy was complemented by efforts to centralize program access to reduce frustration related to navigating multiple platforms related to the program (*e.g.*, consolidated surveys, video visits, and educational materials into the existing patient portal). Additional details on adaptations can be found in the Appendix (Supplemental Table 1).

**Figure 3:**
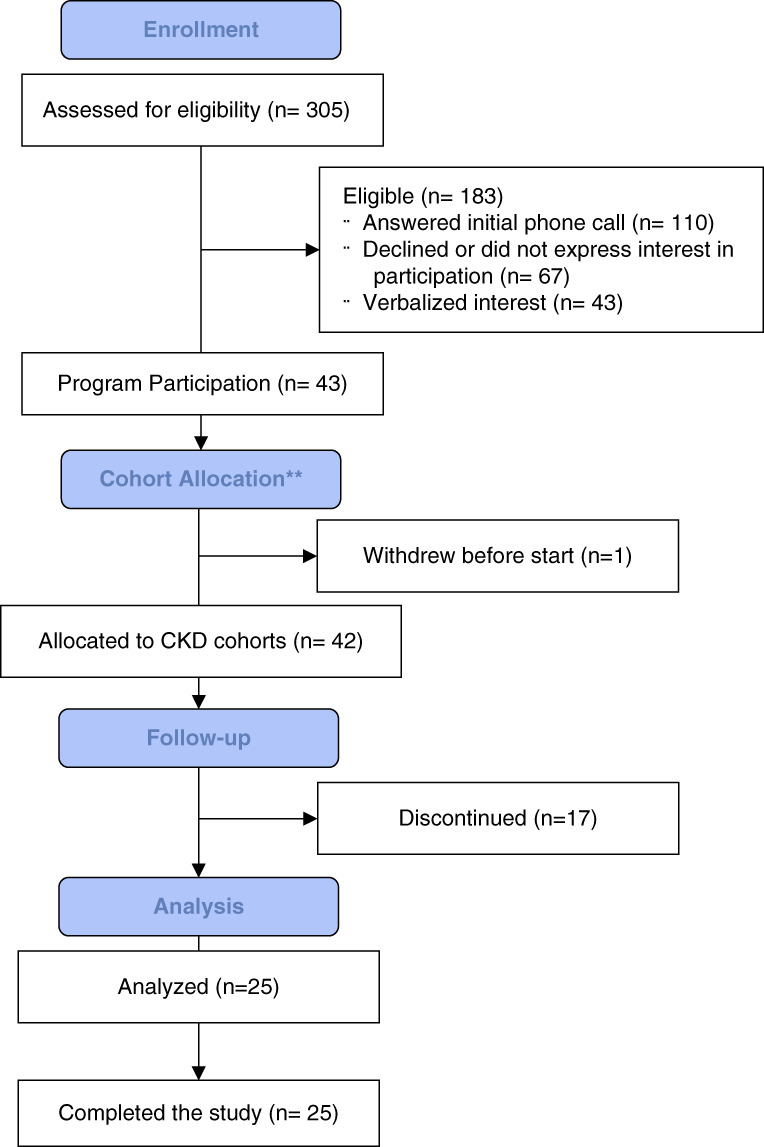
**CONSORT flow diagram.** **Patients with advanced CKD and T2D who were part of the health system's ACO were assigned to cohorts 4 (*n*=21) and 5 (*n*=22), which ran independent sessions. We excluded patients who had other serious non-CKD illnesses, were blind, had severe mental illness, or had limited English proficiency because they were deemed ineligible for the study. ACO, accountable care organization; CONSORT, Consolidated Standards of Reporting Trials.

Of 110 eligible patients who were contacted, 39% (43) were interested in participating. Of those, 58% (25) participated in the 4-month program (Figure 3). Of those, 88% attended half or more of the sessions. The most reported reasons provided for not participating were access-related (*i.e.*, not having access to device to join the virtual visits) and reporting that their condition was already well managed. The mean age of participants was 70.1 years (SD, 8.9), 44% identified as female, 52% as White, 44% as Black, and 64% as married (Table 1). On the basis of a Wilcoxon signed-rank test, baseline and final HbA1c differed significantly (*P* = 0.0176) with a baseline median of 7.05% and a postintervention median of 6.75% (Table 2). A majority of patients had reductions in BP, body mass index, and HbA1c from preintervention to up to 6 months post intervention. Patients were satisfied with the program, with over 90% of the patients who completed the final survey (*n*=12) indicating that they would participate in a CKD telehealth program again and 100% responding that they would recommend the program to others. Patients found the program acceptable and feasible to participate in. Most respondents (66%) agreed or strongly agreed that the virtual visits were easier than normal appointments. Similarly, 75% of patients felt comfortable asking questions, and 83% felt comfortable sharing health information. Additional detail on self-reported health and self-management among patients before and after the program is described in Table 3.

**Table 1:** Participant demographics^a^

Participant Characteristics	*n*=25
Age, mean (SD)	70 (9)
Younger than 65, no. (%)	20 (80)
Older than 65, no. (%)	5 (20)
Female, *no.* (%)	11 (44)
**Race/ethnicity, *No.* (%)**	
White, non-Hispanic	13 (52)
Black, non-Hispanic	11 (44)
Asian, non-Hispanic	1 (4)
**Marital status, *No.* (%)**	
Married	16 (64)
Widowed	0
Divorced	5 (20)
Separated	1 (4)
Never married	3 (12)
**Family history, *no.* (%)**	
CKD	5 (20)
Diabetes	12 (48)
Other chronic conditions	18 (72)

a All patients included have diagnoses of CKD and T2D.

**Table 2:** Wilcoxon signed-rank tests of clinical markers and goals

Outcome	Baseline, Med. (Q1–Q3)	Final, Med. (Q1–Q3)	Δ, Med. (Q1–Q3)	*P* Value
**Attendance**				
Attendance (sessions)	—	7 (6–7)	—	—
Attendance (3 mo), *No.* (%)		5 (20)		
Attendance of 4+ sessions, *No.* (%)		22 (88)		
**Health outcomes**				
Systolic BP, mm Hg	132 (114–142)	122 (118–131)	−2.0 (−12 to 8)	0.4544
Diastolic BP, mm Hg	73.0 (67.0–78.0)	71.5 (64.5–74.0)	−2.0 (−7.5 to 4.5)	0.2970
HbA1c, %	7.05 (6.60–8.30)	6.75 (6.30–7.50)	−0.275 (−1.3 to 0)	0.0176*
GFR, ml/min	35 (33–42)	38 (32–43)	0 (−3 to 9)	0.1674
BMI, kg/m^2^	29.0 (26.8–32.3)	29.2 (25.6–31.6)	−0.45 (−0.8 to 0.10)	0.0522
**Goal progress^a^**				
Goals per patient		2 (1–2)		
Max goal progress, *No.* (%)				
*Total*		51 (100)		
Achieved goal		35 (68.6)		
Made progress		15 (29.4)		
Dropped goal		1 (1.96)		
*Exercise and movement*		22 (43.1)		
Achieved goal		17 (77.3)		
Made progress		4 (18.2)		
Dropped goal		1 (4.55)		
*Food, drink, and diet*		17 (33.3)		
Achieved goal		9 (52.9)		
Made progress		8 (47.1)		
Dropped goal		0 (0.0)		
*Emotions and thoughts*		5 (9.8)		
Achieved goal		4 (80.0)		
Made progress		1 (20.0)		
Dropped goal		0 (0.0)		
*Other goals^b^*		7 (13.7)		
Achieved goal		5 (71.5)		
Made progress		2 (28.6)		
Dropped goal		0 (0.0)		

BMI, body mass index; HbA1c, hemoglobin A1c.

a We recorded the highest progress over the eight sessions, with achieved goal being the highest possible progress, followed by made progress, try again, did not address, and dropped goal.

b Other goals included healthy behaviors (*n*=2), loved ones (*n*=1), personal growth (*n*=2), and sleep and rest (*n*=2). Patients made progress in loved ones and sleep and rest and achieved goals in all others (including for one sleep and rest goal).

**Table 3:** Patient acceptability, self-reported health, and self-management survey results

Survey Measures	Pre (*n*=25)	Post (*n*=12)
**Self-reported health and self-management behaviors**		
GSRH, mean (SD)^a^	2.59 (0.75)	2.79 (0.90)
BP checking^b^, % (*n*)	52.0 (13)	58.3 (7)
Lab results tracking^b^, % (*n*)	80.0 (20)	75.0 (9)
Symptom tracking^b^, % (*n*)	56.0 (14)	75.0 (9)
Emotional management^b^, % (*n*)	60.0 (15)	66.7 (8)
Choosing healthier diet^b^, % (*n*)	76.0 (19)	83.3 (10)
Relaxation^b^, % (*n*)	64.0 (16)	75.0 (9)
Exercise^b^, % (*n*)	76.0 (19)	75.0 (9)
Taken medications not as prescribed^b^, % (*n*)	0.0 (0)	8.3 (1)
Not taken medications^b^, % (*n*)	4.0 (1)	0.0 (0)
Checked blood sugar level^b^, % (*n*)	100.0 (25)	100.0 (12)
Missed visits^b^, % (*n*)	8.0 (2)	0.0 (0)
**Program acceptability and satisfaction, % (*n*)**		
I was comfortable completing the group without in person contact^c^	—	91.7 (11)
I was comfortable with being on camera during the group^c^	—	83.3 (10)
I was comfortable using the video chat functions^c^	—	83.3 (10)
The provider took time to create my personal health plan with me^c^	—	100.0 (12)
My personal health plan included what is important to me^c^	—	100.0 (12)
The program helped me to create goals that I could work on during the group^c^	—	91.7 (11)
I have made progress towards achieving my goals^c^	—	66.7 (8)
I gained valuable information from the group leader(s)^c^	—	100.0 (12)
There was adequate time to ask questions during the group^c^	—	100.0 (12)
It was easy for me to state my concerns and ask questions during the group sessions^c^	—	91.7 (11)
I gained valuable information from listening to other patients' questions and responses^c^	—	91.7 (11)
I benefited from the support I received from other group members^c^	—	75.0 (9)
The technology I used to participate in each group session was effective^c^	—	91.7 (11)
The technology I used to participate in each group session was easy to us^c^	—	91.7 (11)
I feel my medical information is secure in the group setting^c^	—	91.7 (11)
I feel that things I shared during the group were kept private^c^	—	100.0 (12)
The program helped me to improve my health and well-being^c^	—	100.0 (12)
My medication reminder plan I have established or shared with my health coach has helped me to take my medications as prescribed by my doctor^c^	—	83.3 (10)
I would participate in a telehealth CKD program again^c^	—	91.7 (11)
I would recommend a telehealth CKD program to other patients^c^	—	100.0 (12)
I felt like the group leader(s) cared about my well-being during the group^c^	—	100.0 (12)
The video appointment was much easier than my normal appointments^c^	—	66.7 (8)
I felt comfortable asking my doctor questions through my online visit^c^	—	75.0 (9)
I am comfortably sharing or entering my health information electronically^c^	—	83.3 (10)
**Suggested changes to the program, % of responses (*n*)**	—	
Length	—	15.38 (2)
Group discussion	—	7.69 (1)
I would not change anything	—	76.92 (10)

a Response options included 1: poor, 2: fair, 3: good, 4: very good, 5: excellent.

b Response options included not at all, a little, most of the time, and all of the time. Recorded percent represents where patients responded with most of the time or all of the time.

c Response options included disagree, neutral, agree, and strongly agree. Recorded percent represents where patients responded with agree or strongly agree.

## Discussion

An evidenced-based, onsite personalized group visit care model for patients with stage 3b or 4 CKD and T2D was adapted for telehealth delivery and studied in a pilot trial. The telehealth adaptation required significant changes to *periphery* intervention components, including delivery modality (virtual versus in-person) and educational materials (digital versus paper) and content unique to the needs of patients with diabetic kidney disease while preserving *core* intervention components associated with improving outcomes. To this end, we prospectively tracked adaptations to help ensure that the virtual delivery would not compromise the *core* components of the onsite model. Thus, implementation strategies focused on integrating the adapted program with existing telehealth infrastructure, addressing barriers to participation, and emphasizing the importance of digital literacy and equity considerations by providing patients with additional support.^33^ Integrations with patient portal systems and self-management education materials were made to leverage existing infrastructure in tandem with telemedicine visits. While we made these adjustments in response to the coronavirus disease 2019 pandemic, the virtual group visit modality may be more accessible and preferred for many patients as a complementary program to clinical care.

We found that many patients were interested in a program like this (39% verbalized interest in participating), but due to scheduling and other social and economic barriers to participation, only 58% completed the program. Behavioral and pharmacological intervention initiation and adherence is a persistent challenge. We observed a similar dropout rate as a previous study that used in-person sessions for patients with T2D^24^ and a higher drop-out rate when compared to an in-person T2D shared medical appointment trial which observed a 78.4% overall attendance rate, with 44% of patients attending all sessions.^14^ Our study may have experienced lower uptake because of not providing incentives to participate when compared with previous trials. Challenges with uptake are consistent with a broader literature on the implementation of group visits or shared medical appointments which highlight patient attendance as a barrier.^34^ Our study and previous research suggest a need to design more effective strategies to improve enrollment and retention, that is, enhancing the effectiveness of initial eligible patient contact, providing incentives to alleviate economic barriers, providing needed equipment, and offering more flexible schedules to enable virtual visits. Future research should focus on identifying strategies to promote uptake among patients and caregivers who could benefit from improved self-management skills. Among patients who completed the program (*n*=25), 88% (*n*=22) attended a majority of the group sessions, and over half attended seven of the eight sessions. Patients who completed the program reported high levels of satisfaction with 100% reporting they would recommend a telehealth CKD program postparticipation. Because we experienced relatively large groups at the sessions (12–13 participants), future research should explore whether there is a threshold of attendees beyond which the effectiveness or feasibility could be compromised. This is a critical aspect of implementation but was not explicitly evaluated in this pilot.

There are several limitations to this research that should be considered. First, we collected postprogram satisfaction data from 12 of the patients, who may not have held views that are representative of all eligible patients. Moreover, it is important to consider that patients with limited digital literacy may encounter challenges in successfully engaging with the program. Although we introduced a cofacilitator to address technological difficulties, additional policies and strategies to ensure equitable access and uptake are required.^35,36^ Further research is needed on strategies to overcome barriers to initiation or ongoing engagement with these types of self-management programs. Hence, it is crucial for future implementation efforts to prioritize strategies aimed at enhancing patient engagement and uptake, given the observed challenges encountered in this study.

Almost all patients either achieved or made progress on self-reported goals during the intervention. Participants who completed the program demonstrated statistically significant improvements in HbA1c up to 6 months postprogram participation; the small sample size raises concerns about the validity of this observed significance. No significant changes in eGFR were observed. In addition to the small sample size, the lack of a comparator group and short duration of the follow-up limited the extent to which conclusions on clinical effectiveness can be made. Furthermore, we abstracted preprogram values on basis of the most recent clinical data for medical charts. Therefore, baseline clinical values may not have reflected actual values at the time immediately before program participation. In addition, CKD tends to progress gradually, and the goal of treatment is generally to slow or halt eGFR decline, not to yield an absolute eGFR improvement.^37^ Thus, an intervention halting that progression would be clinically meaningful. In addition, we caution against generalizing these findings to other populations or contexts. Future research including an active comparator group would provide additional insight into the effectiveness of the intervention.

In addition to future research on effectiveness, there is a need to validate care models that support self-management among adults who are managing multiple complex conditions. Group visit models, as described herein, may be useful for the unique, intersecting social and medical needs of patients and their caregivers who struggle to effectively manage their CKD by providing the benefits of education, coaching, and peer support. Programs like these could be especially beneficial in a value-based reimbursement system where there are financial incentives to use nonclinical health coaches and technologies to avoid costly hospitalizations or emergency department visits. The studies described herein include implementation approaches that could accelerate the translation of novel telehealth into diverse clinical contexts.

## Supplementary Material

SUPPLEMENTARY MATERIAL

## Data Availability

All data are included in the manuscript and/or supporting information.

## Supplemental Material

Supplemental Table 1. Program adaptations, intervention refinements, and modifications organized by the Framework for Reporting Adaptations and Modifications–Expanded (FRAME).
