# Supplementary material for: Adaptation to Telehealth of Personalized Group Visits for Late Stage Diabetic Kidney Disease
Source: Kidney360. 2023 Nov 14;4(12):1708–16. doi: 10.34067/KID.0000000000000301 (PMC10758514; doi:10.34067/KID.0000000000000301)
Supplement: SUPPLEMENTARY MATERIAL [file kidney360-4-1708-s001.pdf]

APPENDIX

Supplementary Table 1. Program Adaptations, Intervention Refinements, and Modifications Organized by the Framework for Reporting Adaptations and Modifications-Expanded (FRAME)

Adaptation Area of Focus and Original Intervention Component Description	*Rationale, Planning, and Stakeholder Determination*	*Adaptations and Modifications: What, When, How, and Level of Delivery*	*Nature of Modification and Extent to Which Fidelity Is Preserved*
Eligibility, recruitment, and outreach to eligible patients had to be adapted from the original strategies used for the in-person program. Original recruitment mechanisms relied on clinic advertising, provider referrals during visits, and a direct mail campaign to eligible patients.	Initial piloting of the recruitment and outreach process revealed several insights:<br>• Patients without a patient portal account experienced navigating and accessing the program.<br>• Low digital literacy may drive attrition and poor retention.<br>• Minimal outreach to patient during recruitment and enrollment may lead to poor initial engagement.	Patients meeting initial eligibility criteria, who accessed their patient portal to view the program invitation letter, received a follow-up call for screening and to address anticipated technology barriers including<br>• Ensuring patient had reliable internet connection and video/audio capable device (desktop computer, laptop computer) to access the virtual group sessions.<br>• Providing instructions on how to access the online sessions and verbalizes confidence in being able to navigate a web browser.<br><br>Increased outreach and education during enrollment including:<br>• automated notifications informing on group visit scheduling and reminders<br>• a welcome message and additional information regarding intervention materials and surveys to complete via MyChart from their health coach.	The adaptations sought to improve recruitment, lower attrition, and increase retention. To do so, adaptations included a focus on improving technology literacy enabling access and full participation in the telehealth intervention. The changes retained fidelity to the intervention while refining recruitment and enrollment processes to support program engagement without compromising or altering components associated with potential benefit.
Group and individual visit format was changed to incorporate telehealth and patient portal technologies. The original intervention was conducted exclusively in-person.	Adaptations were based on the following factors and justifications:<br>• Preserve intervention components related to motivating patients to focus on their health and providing a social support opportunity<br>• Consolidate access and reduce patients having to navigate between multiple platforms in the program.<br>• Centralize documentation and data entry to ensure efficiency	Adaptations were made to the original in-person intervention for the group visit model for telehealth as follows:<br>• Existing EHR capabilities and integrated extensions (e.g., patient portal and reminder systems) were used for group video visit scheduling, surveys, messaging, and compliant patient access.<br>• Programmatic educational materials were integrated within patient portal systems<br>• Streamlined process for documentation and data collection	The full programmatic digital integration of the program retained fidelity of the intervention and demonstrated how it may be implemented as an element of standard of care.
Self-care educational materials design and access were originally delivered exclusively via printed participant notebook.	Adaptions of the original in-person intervention's self-care educational materials to the group visit model for telehealth were made based on the following factors:<br>• The full programmatic digital integration of the program permitted scheduled release of patient educational material.<br>• A digital patient notebook could integrate with existing EHR and patient portal technologies and simplify scaling the program.	Adaptations were made to the original self-care educational materials for the group visit model for telehealth as follows:<br>• Patients were provided access to an electronic participant notebook through MyChart Digital Care Tab upon being scheduled into the initial group visit.<br>• Electronic version of notebook included embedded links to expand curriculum and enhance learning experience.<br>• Patients continued to receive a printed, hard copy of the notebook.	The digital integration of the patient educational materials enhanced the scope of content and patient learning experience while retaining the fidelity of the instruction provided.
Staffing was changed from the original intervention due to the clinical complexity of the	Adaptations to staffing in the original in-person intervention to the group visit model for telehealth were made based on the following factors and justifications:	Adaptations were made to the original staffing model for the group visit model for telehealth as follows:<br>• Provider involvement with PHP retained by requiring completion of a health risk-assessment, advising on	The adaptations improved management of staffing resources and program efficiencies. These changes were made to retain fidelity

patient population and logistical challenges related to a virtual group visit format.	• Provider 1:1 telehealth encounter with each patient between group sessions and for PHP processes was not feasible due to provider time constraints<br>• Provider presence at each group visit was not feasible due to provide time and resource constraints.<br>• Due to the complexity of the patient population in chronic disease management, facilitator (i.e., health coach) was required to have a clinical licensure.<br>• Patients experiences technical difficulties or is in need of tailored instruction or support during group sessions to navigate technology.	therapeutic plan and indicating health goals for his/her patient. This process was simplified by auto-populating health goals based on guideline concordant recommendations for CKD self-management.<br>• In lieu of provider 1:1 encounter, credentialed facilitator met 1:1 with patient to establish PHP<br>• Facilitator required to hold a national board health coach certification or integrative medicine health coach certification and clinical licensure (e.g., RN, RD, etc.)<br>• Co-facilitator role is a non-clinician role created to:<br>○ assist patient with technical difficulties and navigate the technology during group visits,<br>○ assist in patient 1:1 goal progress virtual room, breakout sessions,<br>○ monitor virtual room to alert facilitator of patient comments or questions, and<br>○ support patient access to all program materials (e.g., participant notebook, surveys).	of intervention while addressing provider involvement and the complexity of the patient population.<br><br>The adaptation to the co-facilitator role provided additional support to the facilitator and patient to enable more efficient use of time and improve the patient experience thereby preserving the fidelity of the intervention in a virtual environment.
Patient engagement techniques were adapted from the original, in-person intervention to simulate the discussion, social support, and problem solving that characterized sessions in the original program. This included didactic sessions and interactive activities to support PHP goal setting and action steps such as using the printed notebook to record and track goals / action steps and goal progress check-ins at each session to share progress with the group	Adaptions of the original in-person intervention to the group visit model for telehealth were made based on the following factors and justifications:<br>• Patients often times have individual questions and concerns<br>• PHP process is individualized and requires coaching surrounding topics and situations that are unique to an individual patient (and caregiver).<br>• Nutrition is a topic of significant interest and required specialized expertise.<br>• Patients often had difficulty in learning how to create a SMART goal despite instruction provided.<br>• Peer support and problem solving to help achieve goals was a strength of the group visit modality.	Adaptations were made to the original in-person intervention for the group visit model for telehealth as follows:<br>• The group visit sessions were reduced in length from 90 minutes to 60 minutes and 1:1 coaching sessions were added to address tailored support that was previously conducted in a group.<br>• Health coach (i.e., facilitator) is available for 1:1 check-in visits as needed<br>• Registered dietician involved with nutrition session alongside the health coach<br>• Each patient was asked to report on goal progress as each group visit and "breakout rooms" were created to track and report on each patient's goals<br>○ Patient assigned to facilitator or co-facilitator for tracking consistency throughout program<br>○ Leverages time for goal progress tracking and reporting at each session<br>○ Allows more 1:1 check in time with each patient, particularly in managing time with larger cohorts<br>○ Patients tended to share more in breakout sessions	The provision of facilitators to lead visits and support patients 1:1 as appropriate improved patient education, engagement, and personalization.<br><br>The adaptation to establishing, tracking, and reporting on shared SMART goals provided additional support to the patient while optimizing use of group session time.
